# Bispecific NK-cell engager targeting BCMA elicits stronger antitumor effects and produces less proinflammatory cytokines than T-cell engager

**DOI:** 10.3389/fimmu.2023.1113303

**Published:** 2023-04-11

**Authors:** Xinghui Xiao, Ying Cheng, Xiaodong Zheng, Yuhang Fang, Yu Zhang, Rui Sun, Zhigang Tian, Haoyu Sun

**Affiliations:** ^1^ Hefei National Research Center for Physical Sciences at Microscale, The CAS Key Laboratory of Innate Immunity and Chronic Disease, School of Basic Medical Sciences, Division of Life Sciences and Medicine, University of Science and Technology of China, Hefei, China; ^2^ Institute of Immunology, University of Science and Technology of China, Hefei, China; ^3^ Hefei TG ImmunoPharma Corporation Limited, Hefei, China

**Keywords:** NK-cell engager, T-cell engager, tumor immunotherapy, antitumor, proinflammatory cytokines

## Abstract

Bispecific antibodies have attracted more attention in recent years for the treatment of tumors, in which most of them target CD3, which mediates the killing of tumor cells by T cells. However, T-cell engager may cause serious side effects, including neurotoxicity and cytokine release syndrome. More safe treatments are still needed to address unmet medical needs, and NK cell-based immunotherapy is a safer and more effective way to treat tumors. Our study developed two IgG-like bispecific antibodies with the same configuration: BT1 (BCMA×CD3) attracted T cells and tumor cells, while BK1 (BCMA×CD16) attracted NK cells and tumor cells. Our study showed that BK1 mediated NK cell activation and upregulated the expression of CD69, CD107a, IFN-γ and TNF. In addition, BK1 elicited a stronger antitumor effect than BT1 both *in vitro* and *in vivo*. Combinatorial treatment (BK1+BT1) showed a stronger antitumor effect than either treatment alone, as indicated by *in vitro* experiments and *in vivo* murine models. More importantly, BK1 induced fewer proinflammatory cytokines than BT1 both *in vitro* and *in vivo*. Surprisingly, BK1 reduced cytokine production in the combinatorial treatment, suggesting the indispensable role of NK cells in the control of cytokine secretion by T cells. In conclusion, our study compared NK-cell engagers and T-cell engagers targeting BCMA. The results indicated that NK-cell engagers were more effective with less proinflammatory cytokine production. Furthermore, the use of NK-cell engagers in combinatorial treatment helped to reduce cytokine secretion by T cells, suggesting a bright future for NK-cell engagers in clinical settings.

## Introduction

In recent years, bispecific antibodies (BsAbs) have been developed rapidly for the treatment of hematologic malignancies. More than 100 formats of BsAbs have entered clinical trials with good efficacy (1). Most BsAbs have been dominated by bispecific T-cell engagers (BiTEs) in preclinical and clinical investigations. The most representative T-cell engager is blinatumomab (2), a BiTE targeted to CD19 and CD3 for B-cell malignancies. Very low doses of blinatumomab lead to elimination of target cells in the blood, bone marrow and liver in non-Hodgkin’s lymphoma patients (3).

T-cell-based engagers can trigger T-cell activation, proliferation, cytokine release and tumor cell killing that bypasses T-cell receptor (TCR) and peptide major histocompatibility complex (pMHC) contact (4, 5). However, despite these promising outcomes, cytokine release syndrome (CRS) and neurological toxicity remain major factors hindering the development of T-cell engagers (6, 7). CRS is mainly caused by proinflammatory cytokines, including tumor necrosis factor (TNF)-α, interferon (IFN)-γ (produced by T cells), interleukin (IL)-1β and IL-6 (produced by myeloid cells) (8, 9), and CRS is more severe during the early stage of administration (4). Furthermore, administration of BiTE also causes severe neurotoxicity; some patients may have neurotoxic symptoms, such as tremors, brain disorders, and seizures, after treatment with blinatumomab (10). In a phase II clinical trial, 189 patients with relapsed or refractory B-precursor acute lymphoblastic leukemia were treated with blinatumomab. The data showed that the most frequent grade 3 or worse adverse events were febrile neutropenia (48 patients, 25%), neutropenia (30 patients, 16%), and anemia (27 patients, 14%). Three (2%) patients had grade 3 CRS. Neurological events of worst grade 3 or 4 occurred in 20 (11%) and four (2%) patients, respectively. Three deaths were thought to be treatment-related (7). Therefore, side effects and safety issues are the major impediments to the application of T-cell engagers.

NK cells, important effector cells of innate immunity, also play an important role in antitumor effects. At present, much evidence has shown that the proportion, infiltration and function of NK cells in patients are closely related to the survival of patients (11). The higher infiltration of NK cells is accompanied by a better clinical effect in solid tumors such as colorectal cancer, breast cancer, clear cell kidney cancer, head and neck cancer and throat cancer (12). Unlike CD8^+^ T cells, NK cells do not express antigen-specific receptors, and their cytotoxicity is controlled through activating and inhibitory receptors on the cell surface. NK-cell engagers mainly activate NK cells by targeting CD16, NKp46, or NKG2D on NK cells (6, 13–18). BiKEs and TriKEs are the main forms of NK-cell engagers. BiKE often has a smaller molecular weight, which facilitates easier penetration into the tumor microenvironment (19). Previous research has shown that CD16xCD33 BiKE significantly enhances degranulation and cytokine production (TNF-α and IFN-γ) against HL-60 cells and endogenous CD33^+^ MDS cells and successfully reverses the immunosuppression of NK cells (20).

B-Cell Maturation Antigen (BCMA), also termed TNFRSF17 or CD269, is a member of the tumor necrosis factor receptor (TNFR) superfamily (21). Ligands of BCMA include B-cell activating factor (BAFF) and proliferation-inducing ligand (APRIL). BCMA is selectively expressed on long-lived bone marrow plasma cells (PCs), not on B-cell precursors, and rarely on other tissues (22). However, BCMA is highly expressed on multiple myeloma cells (23). CD16 is mainly expressed on NK cells and macrophages and is currently known to be one of the most potent activating receptors on NK cells.

In this study, we constructed a two-arm IgG1-based human NK-cell engager, named BK1, against CD16A and BCMA. BK1 was developed for the treatment of BCMA-positive cancers and was designed to redirect NK cells via CD16 to kill BCMA^+^ target cells. Meanwhile, we constructed a T-cell engager, named BT1, with the same configuration. BT1 was developed for the treatment of BCMA-positive cancers and was designed to redirect T cells via CD3. We compared the antitumor effect of BK1 vs. BT1 and found that BK1 was stronger than BT1 when the numbers of NK cells and T cells were equal. Furthermore, BK1 induced significantly less proinflammatory cytokine production than BT1. Combined treatment with BT1 and BK1 showed a stronger antitumor effect than either treatment alone; surprisingly, the use of BK1 in the combined treatment reduced the cytokine production induced by BT1. Our study pointed out that NK-cell engagers should not be underestimated; they may provide even better therapeutic outcomes with fewer side effects (such as CRS). Furthermore, the combined usage of NK-cell engagers and T-cell engagers may help to reduce cytokine production by T cells, suggesting a new therapeutic value of NK-cell engagers.

## Materials and methods

### Ethical approval of the study protocol

The study protocol was approved by the Ethics Committee of the University of Science and Technology of China (Approval No. USTCACUC212301032).

### Mice, cell lines, and reagents

Male or female NSG mice were purchased from SHANGHAI MODEL ORGANISMS (Shanghai, China). Mice were kept in specific pathogen-free conditions according to the National Guidelines for Animal Usage in Research (set by the Chinese government) at the University of Science and Technology of China. Mice between 6 weeks and 8 weeks of age were used.

Cell lines (MM.1S-luc, RPMI 8226) were purchased from the Cell Bank of the Type Culture Collection of Chinese Academy of Sciences (Shanghai, China). The U266 cell line was purchased from Procell (Wuhan, China). The NCI-H929-luc cell line was purchased from SHANGHAI MODEL ORGANISMS (Shanghai, China). NK92 cells were purchased from the American Type Culture Collection (Manassas, USA). 293T cells were purchased from the Cell Bank of the Type Culture Collection of Chinese Academy of Sciences (Shanghai, China). ExpiCHO was purchased from Thermo (Waltham, USA). The cell lines (MM.1S-luc, RPMI 8226, U266, NCI-H929-luc) were cultured at 37°C in an atmosphere of 5% CO_2_ in RPMI 1640 medium (VivaCell, China) supplemented with 10% fetal bovine serum, penicillin (100 U/mL) and streptomycin (100 U/mL). NK92 cells were cultured at 37°C in an atmosphere of 5% CO_2_ in α-MEM (HyClone, Logan, USA) supplemented with 12.5% fetal bovine serum, 12.5% horse serum, 100 units/mL human recombinant IL-2, 10 mM inositol, 0.1 mM 2-mercaptoethanol, 0.02 mM folic acid, penicillin (100 U/mL) and streptomycin (100 U/mL). 293T cells were cultured at 37°C in an atmosphere of 5% CO_2_ in DMEM (VivaCell, Shanghai, China) supplemented with 10% fetal bovine serum, penicillin (100 U/mL) and streptomycin (100 U/mL). ExpiCHO cells were cultured at 37°C in an atmosphere of 8% CO_2_ in ExpiCHO™ Expression Medium (Thermo, Massachusetts, USA).

### Expression and purification of BT1 and BK1

BT1 and BK1 were constructed by recombinant DNA technology and purified from the supernatants of transfected ExpiFectamine™ CHO cells using the ExpiFectamine™ CHO Transfection Kit (Thermo, Massachusetts, USA). The supernatants were then affinity purified with protein A. The molecular weight and purity of BT1 and BK1 were determined by SDS-PAGE.

### Isolation and purification of NK cells and T cells

Blood samples were collected from adults at the Blood Center of Anhui Province (Hefei, China). PBMCs were isolated by centrifugation through a density gradient according to the manufacturer’s instructions (GE Healthcare, Chicago, USA). Negative selection was conducted to purify NK cells and T cells using a MACS kit according to the manufacturer’s instructions (Miltenyi Biotec, Bergisch Gladbach, Germany).

### Binding assay

For the binding assay, tumor cells or effector cells were incubated with various concentrations of BT1 or BK1 for 30 min at 4°C. After washing, tumor cells or effector cells were stained with anti-IgG Fc antibodies for 30 min at 4°C. Then, the positive proportion was detected using flow cytometry.

### Enzyme-linked immunosorbent assay

For binding of a bispecific antibody to antigen, antigens (1 μg/mL) were incubated in each well overnight at room temperature. The wells were blocked for 2 h with blocking buffer and incubated for 2 h with bispecific antibodies (IgG, BT1, or BK1) at serial dilutions. The wells were washed with PBST and incubated for 1 h with HRP-conjugated AffiniPure Mouse Anti-human IgG (BOSTER, Wuhan, China). Then, the cells were incubated with 1-Step™ Ultra TMB-ELISA Substrate Solution (Thermo, Massachusetts, USA) after the wells were washed, followed by stop Solution (Multi Sciences, Hangzhou, China). The absorbance at 450 nm (OD450) was read.

### Quantification of cytokines in cell culture supernatants

Cytokine concentrations were analyzed from supernatants of cytotoxicity assays using the Human Th1/Th2/Th17 CBA Kit (human) Kit (BD, New Jersey, USA) and LEGENDplex™ Human CD8/NK Panel (13-plex) (Biolegend, San Diego, USA) following the manufacturer’s instructions.

### Cytotoxicity assay

RPMI 8226, NCI-H929-luc and MM.1S-luc cells were labeled with CellTrace™ Violet (CTV) (Thermo, Massachusetts, USA) and incubated with PBMCs, purified human NK cells, or purified human T cells at the indicated E:T ratios in the presence of IgG, BT1, BK1 or BK1 combined with BT1 for 6 h or 24 h. For the spontaneous death control, CTV-labeled target cells were cultured alone under identical conditions. After 6 h or 24 h, the wells were harvested, and 7-aminoactinomycin D (7-AAD) (BD, New Jersey, USA) was added before analyses. Samples were mixed thoroughly and analyzed by flow cytometry.

### Flow cytometry

For flow cytometry, CellTrace™ Violet (CTV) was obtained from Invitrogen (C34557, Thermo, Massachusetts, USA). 7-AAD (559925), anti-CD107a (563869), anti-CD45 (557833), anti-CD69 (560711), anti-CD14 (563079) and anti-TNF (559321) were obtained from BD Biosciences (USA); anti-CD56 (362538), anti-CD3 (300328), anti-IFN-γ (506504), anti-TCR α/β (306705), anti-FOXP3 (320113), anti-CD16 (980112) and anti-IgG-Fc (410712) were obtained from BioLegend (San Diego, USA).

For staining of the surface markers, PBMCs were cocultured with tumor cells in the presence of BT1, BK1 or BK1 combined with BT1 (1 μg/mL) for 6 h or 24 h. After incubation, PBMCs were stained for CD25, CD69 or CD107a.

For staining of intracellular cytokines, PBMCs were cocultured with tumor cells in the presence of BT1, BK1 or BK1 combined with BT1 (1 μg/mL) for 6 h or 24 h. After incubation, PBMCs were stained for surface markers, fixed, and permeabilized with fixation buffer according to the manufacturer’s instructions (eBioscience, Massachusetts, USA). The fixed cells were then stained with antibodies against IFN-γ and TNF. All samples were acquired on an LSR-II or Celesta flow cytometer (BD, New Jersey, USA) and were analyzed using FlowJo (TreeStar, Ashland, USA).

### Cell sorting

For cell sorting experiments, human CD56^+^ CD16^-^ NK cells were sorted from human CD56^+^ NK cells using BD Fusion (BD Biosciences, USA).

### NK92-CD16 cell-reconstituted xenogeneic tumor model

NSG mice were injected intravenously (i.v.) with luciferase-expressing MM.1S (MM.1S-luc) tumor cells (1 × 10^6^) on day 0 followed by i.v. of CD16-overexpressing NK92 (NK92-CD16) cells (2 × 10^6^) every 6 days for a total of 4 doses beginning on day 4. Tumor-bearing mice were injected i.v. with BK1 (50 μg) or PBS every 3 days for a total of 8 doses beginning on day 4. In addition, tumor-bearing mice were injected i.p. with IL-2 (50,000 units; Jiangsu Kingsley Pharmaceuticals, Nanjing, China) every 2 days for a total of 11 doses beginning on day 4.

### PBMC-reconstituted or purified effector cell-reconstituted xenogeneic tumor model

NSG mice were injected intravenously (i.v.) with luciferase-expressing NCI-H929 (NCI-H929-luc) tumor cells (2 × 10^6^) on day 0 followed by i.v. of PBMCs (5 × 10^6^ or 1 × 10^7^) or a 1:1 mixture of purified NK and T cells (5 × 10^5^) on day 9. Tumor-bearing mice were injected i.v. with IgG, BK1, and BT1 (50 μg) every 3 days for a total of 3 doses beginning on day 9 combined with IL-2 (10,000 units; Jiangsu Kingsley Pharmaceuticals, Nanjing, China) every 3 days for a total of 3 doses beginning on day 9.

### Cytokine detection in the PBMC-reconstituted xenogeneic tumor model

NSG mice were injected intravenously (i.v.) with luciferase-expressing NCI-H929 (NCI-H929-luc) tumor cells (5 × 10^6^) on day 0 followed by i.v. of PBMCs (2 × 10^7^) on day 11. Tumor-bearing mice were injected i.v. with IgG, BT1, BK1, and BT1+BK1 (50 μg/mouse) on day 11 with IL-2 (10000 units; Jiangsu Kingsley Pharmaceuticals, Nanjing, China). Serum cytokines were detected 6 hours after treatment with a V-PLEX Proinflammatory Panel 1 (human) Kit (LX-K15049D-1, LabEx, Shanghai, China).

### Bioluminescence imaging

Mice were injected i.p. with d-luciferin (15 mg/mL; Gold Biotechnology, St. Louis, USA) at 150 mg/kg bodyweight. Mice were placed into an *in vivo* imaging system (Caliper Life Sciences, Waltham, USA) when fully anesthetized by isoflurane. Luciferase expression was imaged using Spectral *In Vivo* (PerkinElmer, Waltham, USA) and calculated using Living Image (PerkinElmer, Waltham, USA).

**Statement:** For animal studies, Tumor-bearing mice were randomized before administration.

### Statistical analysis

Comparisons between 2 groups were analyzed using the unpaired one/two-tailed Student’s t test. Comparisons between ≥ 3 groups were analyzed using 2-way analysis of variance. Kaplan-Meier analyses and Mantel-Cox tests were used to analyze mouse survival. The results are expressed as the mean ± SEM. P < 0.05 was assumed to be significant in all analyses.

## Results

### Construction and binding specificity of BCMA-specific NK-cell engager (BK1) and T-cell engager (BT1)

To compare the function of T-cell engaging bispecific antibody (T-cell engager) and NK-cell engaging bispecific antibody (NK-cell engager), we constructed BT1 (BCMA×CD3 T-cell engager) and BK1 (BCMA×CD16A NK-cell engager), respectively. BT1 is a two-arm IgG1-based human antibody consisting of two single chain variable fragments (scFvs) and Fc (**Figure S1A**). The purity of BT1 was > 90%, as determined by SDS-PAGE (**Figure S1B**). To ascertain the binding ability of BT1, we detected the binding ability of BT1 to the BCMA extracellular segment and CD3 protein through ELISA. The results indicated that the binding ability of BT1 improved with increasing concentrations (**Figures S1C, D**). Furthermore, BCMA^+^ MM.1S-luc cells and CD3^+^ T cells were incubated with Alexa Fluor 647-labeled BT1, and the proportion of Alexa Fluor 647-positive cells increased with BT1 concentrations, indicating that BT1 binds to these BCMA^+^ target cells and CD3^+^ T cells successfully (**Figures S1E, F**).

**Figure 1:**
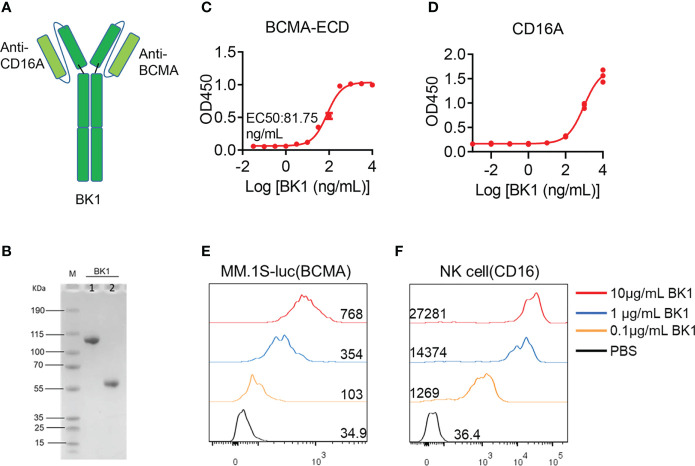
Structural features and binding affinity of BK1 (BCMA×CD16A) bispecific antibody. **(A)** Schematic representation of BK1 (CD16A×BCMA), a two-arm IgG1-based human antibody. **(B)** The molecular weight and purity of BK1 were determined by SDS-PAGE. M: protein marker, 1: Nonreducing electrophoresis, 2: reducing electrophoresis. **(C)** Binding specificity of BK1 with the BCMA extracellular domain protein. **(D)** Binding specificity of BK1 with CD16A protein. **(E)** Binding specificity of BK1 with BCMA^+^ MM.1S-luc cells. BK1 was labeled with Alexa Fluor 647 fluorescein and incubated with BCMA^+^ MM.1S-luc cells at 10μg/mL, 1μg/mL and 0.1μg/mL, respectively, followed by flow cytometric analysis. **(F)** Binding specificity of BK1 with CD16^+^ NK cells. BK1 was labeled with Alexa Fluor 647 fluorescein and incubated with CD16^+^ NK cells at 10 μg/mL, 1 μg/mL and 0.1 μg/mL, followed by flow cytometric analysis. **(C-F)** Data are representative of at least three independent experiments.

In addition, we constructed an NK-cell engager (BK1) with the same structure as BT1, consisting of two scFvs and Fc (**Figure 1A**). The purity of BK1 was > 90%, as determined by SDS-PAGE (**Figure 1B**). To ascertain the binding ability of BK1, we detected the binding ability of BK1 to the BCMA extracellular segment and CD16 protein through ELISA. The results indicated that the binding ability of BK1 improves with increased concentrations (**Figures 1C, D**), and it binds to CD16 mainly through its Fab segment (**Figure S2**). Furthermore, BCMA^+^ MM.1S-luc cells and purified CD16^+^ NK cells were incubated with Alexa Fluor 647-labeled BK1, and the proportion of Alexa Fluor 647-positive cells increased with BK1 concentrations, indicating that BK1 binds to these BCMA^+^ target cells and CD16^+^ NK cells successfully (**Figures 1E, F**). Altogether, these data indicated that BT1 and BK1 have similar structures and sizes and are capable of binding to tumor cells and effector cells.

### BK1 (BCMA×CD16A) specifically kills BCMA^+^ target cells and activates CD16^+^ NK cells

**Figure 2:**
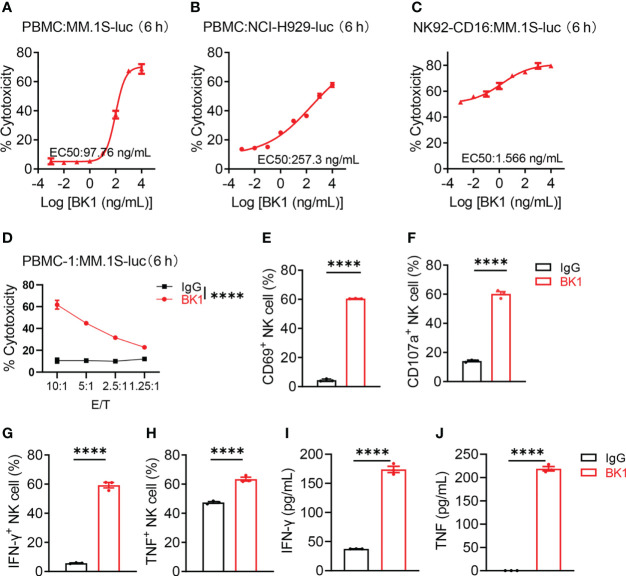
BK1 (BCMA×CD16A) specifically kills BCMA^+^ target cells and activates CD16^+^ NK cells. **(A)** Percentage of tumor cell lysis after coculturing MM.1S-luc cells with PBMCs in the presence of BK1 at an E:T ratio of 10:1 for 6 h. **(B)** Percentage of tumor cell lysis after coculturing NCI-H929-luc cells with PBMCs in the presence of BK1 at an E:T ratio of 10:1 for 6 h. **(C)** Percentage of tumor cell lysis after coculturing MM.1S-luc cells with NK92-CD16 cells in the presence of BK1 at an E:T ratio of 2:1 for 6 h. **(D)** Percentage of tumor cell lysis after coculturing MM.1S-luc cells with PBMCs in the presence of BK1 (1 μg/mL) at the indicated E:T ratios for 6 h. **(E–H)** PBMCs were cocultured with MM.1S-luc cells at an E:T ratio of 10:1 in the presence of IgG (1 μg/mL) or BK1 (1 μg/mL) for 6 h, and CD3^-^CD56^+^ NK cells were assessed for the expression of CD69, CD107a, IFN-γ, and TNF by flow cytometry. **(I, J)** PBMCs were cocultured with MM.1S-luc cells at an E:T-cell ratio of 10:1 in the presence of IgG (1 μg/mL) or BK1 (1 μg/mL) for 6 h, and the levels of secreted IFN-γ and TNF in the supernatants were assessed using a CBA Kit. **(A-J)**: Data are representative of at least three independent experiments. Data were analyzed by Student’s t test or 2-way analysis of variance. ****P < 0.0001.

To compare the effect of BT1 and BK1 on the activation and function of corresponding effector cells, we examined the cytotoxic effect of BT1 and BK1, respectively. PBMCs (culture with 100 IU of IL-2/mL 1640 complete medium) were incubated with MM.1S-luc cells or NCI H929-luc cells in the presence of BK1 for 6 hours, and the results showed that BK1 significantly improved the specific lytic ability of PBMCs against BCMA^+^ tumor cells in a concentration-dependent manner (**Figures 2A, B**, **S3**). Furthermore, the incubation of CD16-overexpressing NK92 (NK92-CD16) cells and MM.1S-luc cells also showed significantly improved lytic ability of NK92-CD16 cells against tumor cells in the presence of BK1 (**Figure 2C**). The lytic ability of effector cells was also increased with an elevated E:T ratio (**Figures 2D**, **S3D–J**). Meanwhile, comparison between the cytotoxicity of CD16^+^ NK cells vs. CD16^-^ NK cells under the effect of BK1 proved that BK1 could only act through CD16^+^ NK cells to exert its anti-tumor function (**Figures S4A-C**). In addition, BK1 significantly enhanced the expression of CD69, CD107a, IFN-γ and TNF-α on CD3^-^CD56^+^ NK cells compared with IgG after coculturing PBMCs with MM.1S-luc cells (**Figures 2E–H**, **S5A-H**). Furthermore, BK1 also significantly enhanced the secretion of IFN-γ, TNF-α, granzyme A, granzyme B, perforin and sFasL (**Figures 2I, J**, **S5I-L**, **S6**). On the other hand, BT1 significantly improved the lytic ability of PBMCs or purified T cells against BCMA^+^ tumor cells (**Figures S7A-F**). Furthermore, BT1 significantly enhanced the expression of CD25, CD69, IFN-γ and TNF-α on CD56^-^CD3^+^ T cells (**Figure S7G**) and the secretion of IFN-γ, IL-2 and TNF-α in the supernatant (**Figures S7H-J**). These data indicated that BK1 was able to activate CD16^+^ NK cells, while BT1 was able to activate CD3^+^ T cells, and both specifically killed BCMA^+^ tumor cells.

### BK1 (BCMA×CD16A) elicits stronger antitumor effects than BT1 (BCMA ×CD3) in xenogenic tumor models

**Figure 3:**
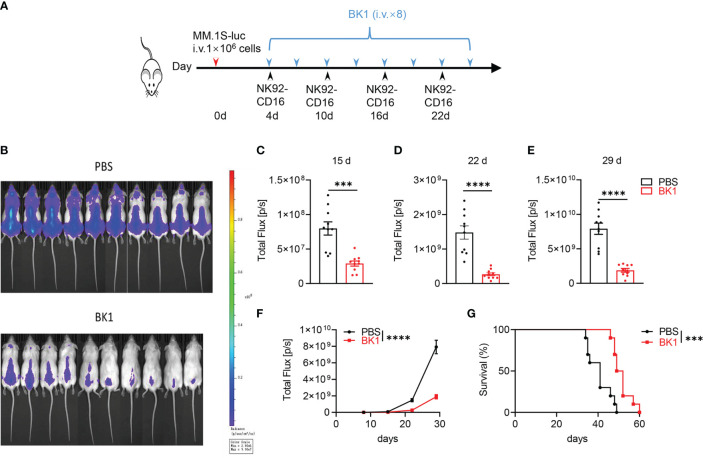
BK1 treatment inhibits tumor growth in an NK92-CD16 cell-reconstituted xenogeneic tumor model. **(A)** Experimental protocol for the tumor model used in **(B–G)**: NSG mice (n = 10/group) were injected intravenously (i.v.) with MM.1S-luc tumor cells (1 × 10^6^) on day 0 followed by intravenous injection of NK92-CD16 cells (2 × 10^6^) on day 4 and every 6 days for a total of 4 times. Tumor-bearing mice were injected intravenously (i.v.) with BK1 or PBS (50 μg/mouse) every 3 days for a total of 8 doses beginning on day 4. **(B)** *In vivo* bioluminescence imaging of mice 22 days after tumor challenge described in **(A)**. **(C–E)** Quantification of luminescence in PBS-treated or BK1-treated mice on days 15, 22 and 29. **(F)** Luminescence in PBS-treated or BK1-treated mice on days 8, 15, 22 and 29. **(G)** Overall survival of PBS-treated and BK1-treated mice. Each symbol **(C–E)** represents an individual mouse. Data were analyzed by Student’s t test **(C–E)**, 2-way analysis of variance **(F)** and the Mantel-Cox test **(G)**, ***P < 0.001, ****P < 0.0001.

Antitumor cytotoxicity is one of the most important functions of bispecific antibodies. We aimed to assess whether BT1 or BK1 improves antitumor function *in vivo*. Here, we established xenogenic models using human NK92-CD16 cells, a purified NK and T-cell mixture, or PBMCs from NSG mice. First, we established xenogeneic models using NK92-CD16 cells to assess the antitumor effect of BK1. These mice were loaded with MM.1S-luc cells and treated with BK1 or PBS (**Figure 3A**). Tumor growth was significantly inhibited by treatment with BK1 (**Figure 3B**). Quantification of luminescence also confirmed significantly delayed tumor growth on days 15, 22 and 29 after BK1 treatment (**Figures 3C–F**). Consistent with the results of tumor growth, xenogeneic mice treated with BK1 also showed significantly improved survival (**Figure 3G**).

**Figure 4:**
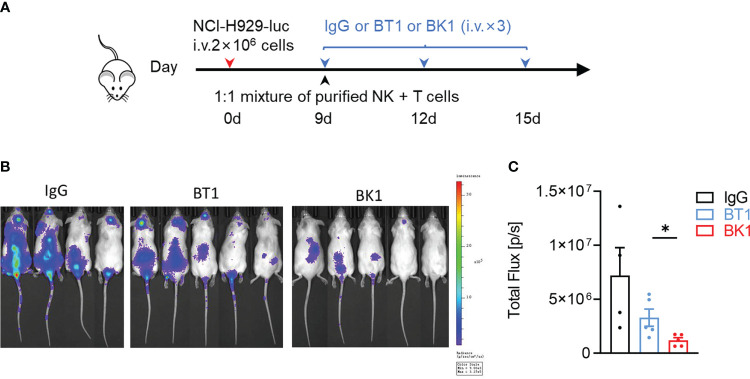
BK1 (BCMA×CD16A) shows a stronger antitumor effect than BT1 (BCMA×CD3) in an NK and T-cell mixture-reconstituted xenogeneic tumor model. **(A)** Experimental protocol for the tumor model used in **(A–C)**. NSG mice were injected intravenously (i.v.) with NCI-H929-luc tumor cells (2 × 10^6^) on day 0 followed by intravenous injection of a 1:1 mixture of purified NK and T cells (5 × 10^5^ each) on day 9. Tumor-bearing mice were injected intravenously (i.v.) with IgG, BT1 or BK1 (50 μg/mouse) every 3 days for a total of 3 doses beginning on day 9. **(B)** *In vivo* bioluminescence imaging of mice 13 days after tumor challenge described in **(A)**. **(C)** Quantification of luminescence in IgG-treated, BT1-treated or BK1-treated mice on day 13. Each symbol represents an individual mouse. **(B, C)** Data are representative of at least three independent experiments. Data were analyzed by Student’s t test *P < 0.05.

**Figure 5:**
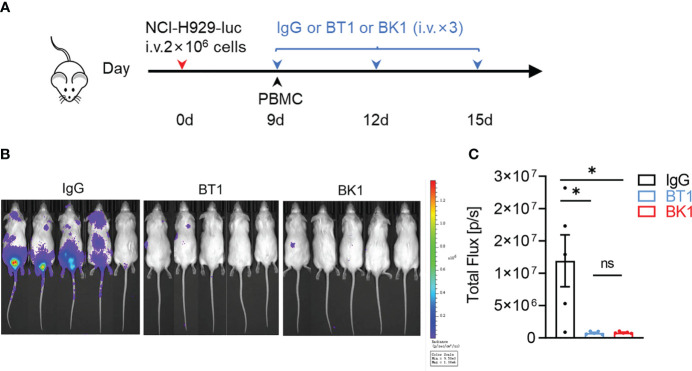
BT1 and BK1 show comparable antitumor effects in a PBMC-reconstituted xenogeneic tumor model. **(A)** Experimental protocol for the tumor model used in **(B, C)**: NSG mice (n = 5/group) were injected intravenously (i.v.) with NCI-H929-luc tumor cells (2 × 10^6^) on day 0 followed by intravenous injection of PBMCs (1 × 10^7^) on day 9. Tumor-bearing mice were injected intravenously (i.v.) with IgG, BT1 or BK1 (50 μg/mouse) every 3 days for a total of 3 doses beginning on day 9. **(B)** *In vivo* bioluminescence imaging of mice 18 days after the tumor challenge described in **(A)**. **(C)** Quantification of luminescence in IgG-treated, BT1-treated or BK1-treated mice on day 18. Each symbol **(C)** represents an individual mouse. **(B, C)**: Data are representative of at least three independent experiments. Data were analyzed by Student’s t test. ns:P>0.05; *P < 0.05.

Since the quantity of T cells (55-65%) is far more numerous than that of NK cells (5-20%) in the peripheral blood, to compare the antitumor effect of BK1 vs. BT1, we used a 1:1 mixture of purified NK cells and T cells to reconstitute the xenogenic models. These mice were then loaded with NCI-H929-luc cells and treated with IgG, BT1 or BK1 (**Figures 4A–C**). The results indicated that BK1 (BCMA×CD16A) significantly inhibited tumor growth and elicited a stronger antitumor effect than BT1 when the numbers of NK cells and T cells were equal. We also established xenogenic models using human PBMCs, and these mice were then loaded with NCI-H929-luc cells and treated with IgG, BT1 or BK1 (**Figure 5A**). The results showed that tumor growth was significantly inhibited by treatment with BT1 or BK1, and BT1 and BK1 showed comparable antitumor effects in these xenogeneic models reconstituted with human PBMCs (**Figures 5B, C**). Altogether, these data indicated that both BT1 and BK1 are effective against tumors, among which BK1 is stronger than BT1 when the numbers of NK cells and T cells are equal. Comparable antitumor effects were achieved when the number of T cells was approximately 3 times greater than the number of NK cells, suggesting that BK1 (BCMA×CD16A) is a stronger antitumor bispecific antibody than BT1 (BCMA ×CD3).

### Combined treatment with BT1 and BK1 elicits the strongest antitumor effects

**Figure 6:**
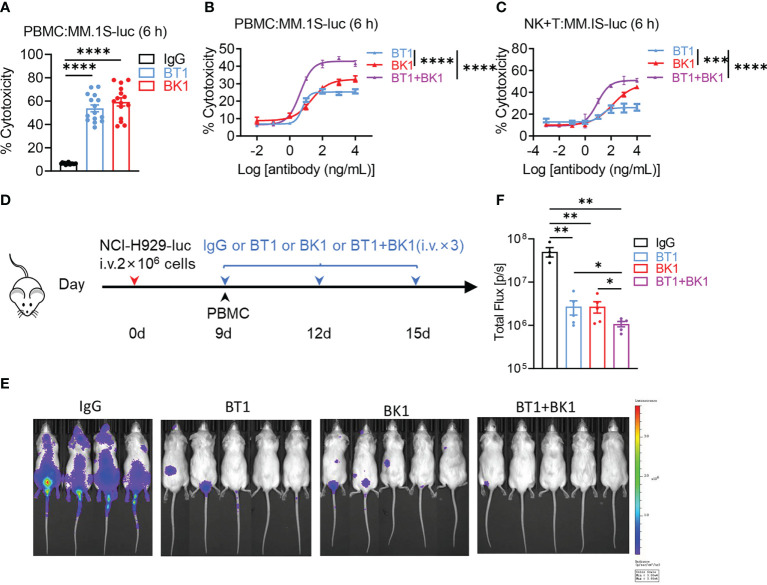
Combined treatment with BT1 and BK1 shows the strongest antitumor effects both *in vitro* and *in vivo* compared with either treatment alone. **(A)** Percentage of tumor cell lysis after coculturing MM.1S-luc cells with PBMCs (n=15) in the presence of BT1 or BK1 at an E:T ratio of 10:1 for 6 hours. **(B)** Percentage of tumor cell lysis after coculturing MM.1S-luc with PBMCs in the presence of BT1, BK1, or BT1 combined with BK1 (at the indicated concentrations) at an E:T ratio of 10:1 for 6 hours. **(C)** Percentage of tumor cell lysis after coculturing MM.1S-luc cells with a 1:1 mixture of purified NK cells and T cells in the presence of BT1, BK1, or BT1 combined with BK1 (at the indicated concentrations) at an E:T ratio of 2:1 for 6 hours. **(D)** Experimental protocol for the tumor model used in **(E, F)**: NSG mice (n = 4 or 5/group) were injected intravenously (i.v.) with NCI-H929-luc tumor cells (2 × 10^6^) on day 0 followed by intravenous injection of PBMCs (5 × 10^6^) on day 9. Tumor-bearing mice were injected intravenously (i.v.) with IgG, BT1, BK1 or BT1+BK1 (50 μg/mouse) every 3 days for a total of 3 doses beginning on day 9. **(E)** *In vivo* bioluminescence imaging of mice 15 days after tumor challenge described in **(D)**. **(F)** Quantification of luminescence in IgG-treated, BT1-treated, BK1-treated or combined-treated mice on day 15. Each symbol **(E, F)** represents an individual mouse. **(A-F)** Data are representative of at least three independent experiments. Data were analyzed by 2-way analysis of variance or Student’s t test. *P < 0.05; **P < 0.01, ***P < 0.001, ****P < 0.0001.

BT1 and BK1 both showed strong antitumor effects in tumor-bearing mice. Next, we wanted to determine whether the combined treatment could elicit stronger cytotoxicity. We used PBMCs to coculture with MM.1S-luc cells in the presence of BK1, BT1 or IgG *in vitro*, and the results indicated that both BT1 and BK1 significantly improved the cytotoxicity after 6 hours of coculturing, in which BK1 (BCMA×CD16A) showed stronger cytotoxicity against target cells than BT1 (BCMA ×CD3) (**Figures 6A, B**). We further assessed the cytotoxicity of BK1, BT1 or BK1 combined with BT1 *in vitro*, and the results indicated that combined treatment significantly improved the cytotoxicity of PBMCs against MM.1S-luc cells after 6 hours of coculturing compared to using either treatment alone (**Figure 6B**). To rule out the influence of other immune cells, we purified NK cells and T cells and cocultured a 1:1 mixture of purified NK and T cells with MM.1S-luc target cells for 6 hours. The results also showed that combined treatment significantly improved the cytotoxicity of the mixed effector cells compared to using either treatment alone (**Figure 6C**).

In addition to the *in vitro* analysis, we also established xenogenic models using human PBMCs with NSG mice to assess the antitumor effect of the combined treatment *in vivo* (**Figure 6D**). Bioluminescence imaging of mice 15 days after tumor challenge showed that BT1 or BK1 alone could reduce tumor growth, while combined treatment with BT1 and BK1 further delayed tumor growth with the strongest antitumor effect (**Figures 6E, F**). Altogether, these data indicated that combining BT1 and BK1 improved the cytotoxicity of effector cells *in vitro* and restrained tumor growth *in vivo*, suggesting that the maximum antitumor effect could be achieved by using combinatorial treatment.

### BK1 induces fewer proinflammatory cytokines and reduces cytokine production by BT1 in the combined treatment

**Figure 7:**
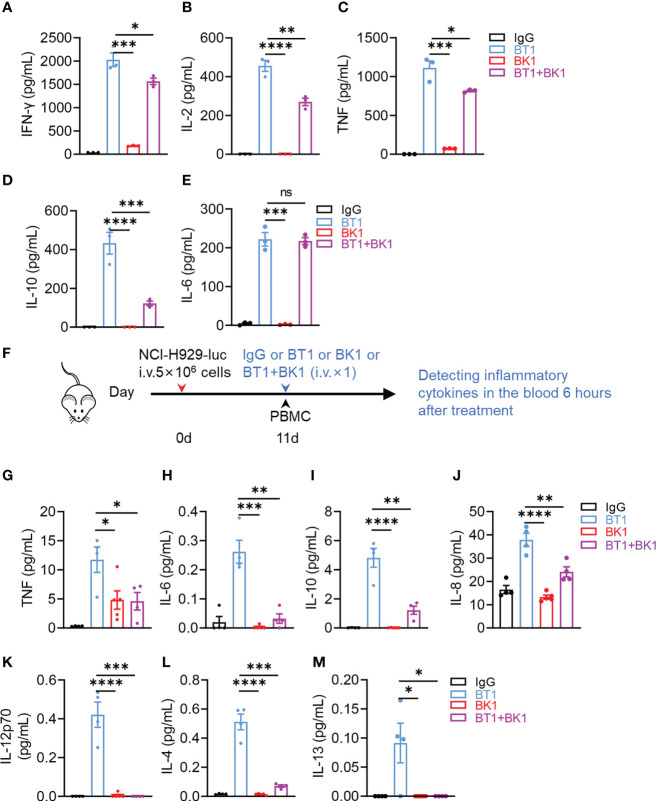
BK1 induces less proinflammatory cytokine production and reduces cytokine secretion by BT1 in the combined treatment. **(A-E)** PBMCs were cocultured with MM.1S-luc cells at an E:T-cell ratio of 10:1 in the presence of IgG, BT1, BK1 or BT1 combined with BK1 (1 μg/mL) for 24 h, and the levels of secreted **(A)** IFN-γ, **(B)** IL-2, **(C)** TNF, **(D)** IL-10 and **(E)** IL-6 in the supernatants were assessed using a CBA Kit. **(F)** Experimental protocol for the cytokine model used in **(G–M)**: NSG mice (n = 5/group) were injected intravenously (i.v.) with NCI-H929-luc tumor cells (5 × 10^6^) on day 0 followed by intravenous injection of PBMCs (2 × 10^7^) on day 11. Tumor-bearing mice were injected intravenously (i.v.) with IgG, BT1, BK1 or combined treatment (50 μg/mouse) on day 11. Inflammatory cytokines in the blood were assessed 6 h following treatment. **(G-M)** Levels of secreted **(G)** TNF, **(H)** IL-6, **(I)** IL-10, **(J)** IL-8, **(K)** IL-12p70, **(L)** IL-4 and **(M)** IL-13 were assessed using a V-PLEX proinflammatory Panel 1 kit. **(A-M)**: Data are representative of at least three independent experiments. Data were analyzed by Student’s t test. ns:P>0.05; *P < 0.05; **P < 0.01; ***P < 0.001; ****P < 0.0001.

Cytokine storms and neurotoxicity are obstructive factors for immunotherapy. To evaluate the potential risks of BT1 and BK1, the cytokine levels in the supernatant were determined after coculture of PBMCs and MM.1S-luc cells for 24 hours in the presence of IgG, BK1, BT1 or BK1 combined with BT1 *in vitro*. The results indicated that BT1 induced significantly higher levels of cytokine secretion, including IFN-γ, IL-2, TNF, IL-10 and IL-6, than BK1 (**Figures 7A–E**, **S8**), in which IL-6, TNF and IL-10 are strong inducers of cytokine storms. Interestingly, significantly lower levels of cytokines, including IL-2, IL-10 and IL-6, were observed in the supernatant of the combined treatment compared with that of BT1, suggesting that the combined usage of BT1 and BK1 reduces cytokine secretion by BT1. To explore the underlying mechanism, we evaluated the PBMC composition after 24 hours of co-culture. The percentage of regulatory T cells and monocytes remained the same after treatment with BT1, BK1 or BT1 combined with BK1 (**Figures S9A, B**). Interestingly, the percentage of T cells decreased after BT1 treatment but recovered after BK1 or BT1 combined with BK1 treatment, suggesting that BK1 could enhance the proliferation of T cells (**Figure S9C**). We further used CTV-tagged PBMCs co-cultured with tumor cells in the presence of BT1 or BT1 combined with BK1 for 3 days to evaluate the proliferation of T cells. As shown in **Figures S9D-F**, combinatorial treatment resulted in higher T-cell proliferation than BT1 treatment alone.

To mimic the *in vivo* microenvironment, we constructed a mouse model of cytokine release syndrome to assess cytokine secretion in the blood serum of tumor-bearing mice. NSG mice were injected intravenously with NCI-H929-luc tumor cells on day 0 followed by intravenous injection of PBMCs on day 11. Tumor-bearing mice were then injected intravenously with IgG, BT1, BK1 or combined treatment on the same day followed by cytokine detection 6 hours later (**Figure 7F**). The results showed that treatment with BK1 induced fewer proinflammatory cytokines, including TNF, IL-6, IL-10, IL-8, IL-12p70, IL-4 and IL-13, than treatment with BT1 (**Figures 7G–M**). These cytokines are closely related to cytokine release syndrome from immunotherapy (24, 25). Surprisingly, combined treatment with BT1 and BK1 *in vivo* induced fewer cytokines than BT1 (**Figures 7G–M**), suggesting that BK1 somehow reduces the cytokine secretion induced by BT1. Altogether, these data indicated that NK-cell engager was safer than T-cell engager with less proinflammatory cytokine production. In addition, BK1-activated NK cells somehow reduced excessive cytokine production by BT1-activated T cells, suggesting that not only NK-cell engager results in less proinflammatory cytokine production but also helps to reduce the proinflammatory cytokines secreted by T cells.

## Discussion

T-cell-based immunotherapy has made impressive progress in the treatment of tumors; however, some concerns remain to be solved. For example, the percentage of patients who can respond to T-cell-based immunotherapy remains below 30%. Previous studies have indicated that T-cell-based immunotherapy is not effective against cold tumors (26). Therefore, it is urgent and necessary to find new therapeutic methods independent of antigen processing and presentation to overcome tumor resistance in T-cell-based immunotherapy. NK cells can detect and kill tumor cells with stochastic combinations of activating receptors and inhibitory receptors independent of antigen and HLA (27). The net balance of activating and inhibitory signals gives rise to either response or tolerance of the target cells (27), suggesting that NK-cell-based immunotherapy holds great promise in the treatment of cancer (28–31).

Currently, NK-cell engagers activate NK cells by targeting CD16, NKG2D or NCR on the surface of NK cells to mediate the lysis of tumor cells (6, 17, 32, 33). CD16-NK-cell-based engagers are the most common type of NK-cell engagers. Several studies have shown that CD16-NK-cell-based engager can mediate the upregulation of CD69, CD107a, TRAIL, IFN-γ and TNF-α in NK cells, promoting the activation and proliferation of NK cells and enhancing the ability of NK cells to kill tumors (13–15). At present, most CD16-NK-cell engagers have BiKE and TriKE configurations without Fc segments, with a short half-life and poor stability with ease of aggregation (34). In our study, we designed an IgG-based NK-cell engager BK1 (CD16A×BCMA) and found that BK1 mediates the activation of NK cells and enhances the anti-tumor efficacy of NK cells.

The cellular immune system, which includes T cells and NK cells, plays a major role in suppressing tumors. However, there are currently few studies evaluating the antitumor abilities of NK cells and T cells in the same system. Melanie Märklin et al. constructed bispecific NKG2D-CD3 and NKG2D-CD16 fusion proteins and found that the NKG2D-CD16 fusion protein mediates higher killing efficiency for 2-8 hours. In addition, the NKG2D-CD3 fusion protein mediates PBMCs to produce higher levels of IFN-γ, granzyme B and perforin than the NKG2D-CD16 fusion protein (35). To compare the antitumor effect of NK cell engager and T-cell engager, we constructed T-cell engager BT1 based on CD3 and BCMA with the same configuration as BK1. BT1 activated T cells against tumor cells and was effective against tumors. The cytotoxicity of BT1 was comparable to that of BI 836909 (BCMA×CD3) (36) reported by S Hipp and EM801 (BCMA×CD3) (37) reported by Anja Seckinger. We found that BK1 significantly inhibited tumor growth and elicited a stronger antitumor effect than BT1 in an *in vivo* model. In addition, our study also showed that combinatorial usage of BT1 and BK1 elicited a stronger antitumor effect than using either one alone. Indeed, NK-cell-induced tumor cell death can increase the release of tumor antigens that in turn enhance T-cell antigen presentation levels and T-cell responses to cancer (38). The cytotoxicity of tumor cells by NK cells results in the release of tumor antigens to trigger an adaptive immune response (39). Meanwhile, our study indicated that BK1 (BCMA×CD16) was able to enhance T-cell proliferation in the combined treatment of BT1 and BK1.

Stimulating immune cells stimulate immune responses and cytokine release, whereas overstimulating immune cells might lead to cytokine release syndrome (40, 41). Cytokine release syndrome is mainly due to the activation of lymphocytes or monocytes to produce cytokines such as TNF, IL-6, and IL-1β, which then cause a severe systemic inflammatory response (9, 24, 42, 43). Cytokine release syndrome is positively correlated with efficacy; however, the balance between efficacy and side effects is still a challenging issue (25, 44, 45). Currently, cytokine release syndrome is the most common toxicity associated with T-cell-based immunotherapy (for example, CAR T-cell therapy, T-cell engager, etc), with an incidence of 42–100% (46). NK cells, on the other hand, produce fewer cytokines than T cells (47). Andrew G. Polson et al. demonstrated that an NK cell engager (BCMA×CD16A) exhibited minimal cytokine release and no RO7297089-related mortalities or adverse events in cynomolgus monkeys (33). In our study, we compared cytokine production of BK1 vs. BT1 in the same system, and we found that BT1 induced higher levels of TNF, IL-6, IL-8, IL-10 and IL-12p70 than BK1 both *in vitro* and *in vivo*. Among these cytokines, IL-6, TNF, IL-10 and IL-8 are very strong inducers of cytokine storms (46, 48–50). In addition, BT1 also induced higher levels of IL-4 and IL-13, which are Th2 response cytokines. IL-4 is considered a master Th2 switch driving the generation of other proallergic cytokines, such as IL-5 and IL-13 (51,) and participates in the migration of Th2 cells and eosinophils to the inflamed site (51). IL-13 is a driver of COVID-19 severity, and neutralization of IL-13 reduces the disease in a mouse model (52). Our study suggests that NK-cell engagers have a lower risk of cytokine storms than T-cell engagers. It is worth noting that the detected level of cytokines was very low *in vivo*. In the study by Gabrielle Leclercg and Lauric Haber et al., the human cytokine levels detected were less than 50 pg/mL in the serum of a PBMC-reconstituted xenogenic tumor model (53, 54). Subsequent evaluation of the toxicology and safety of BK1 vs. BT1 in monkeys is required to reconfirm our results in mice.

Surprisingly, we also found in our study that BK1 was able to reduce cytokine production of BT1 in the combined treatment. The reason for this may be that NK cells may regulate T cells in unknown ways. Studies in microbial infections have demonstrated that NK cells may kill T cells to prevent overactivation of T cells and to avoid detrimental consequences of excessive inflammation (55–57). Furthermore, activated NK cells may alleviate autoreactive T-cell immunity in viral infection (57). The regulatory role of NK cells has also been reported in human autoimmune diseases, and impaired NK cell activity is closely related to the systemic onset of juvenile rheumatoid arthritis and nonobese diabetes (58, 59). On the other hand, the reason for this may be due to the competition between NK cells and T cells for binding to tumor cells, leading to fewer T cells being activated and fewer cytokines being produced. Overall, our study points out that the combined use of NK-cell engagers and T-cell engagers not only improves the antitumor effect but also lowers cytokine release from T-cell engagers, showing the potential of the combined use of both.

However, there are few limitations for this study. First, BK1 could not mediate NK cell proliferation or survival, therefore, IL-2 was used to the culture of PBMCs, NK cells or T cells to maintain the survival of these cells, which may also partially activate NK cells and T cells. Second, treatment with BT1 or BK1 did not eliminate tumor cells, but only inhibited tumor cell growth *in vivo*. Whether increasing the dose of antibody or effector cells can eliminate tumor cells and further prolong the survival requires further study. Third, combination therapy mediated more killer cells to the tumor site with better anti-tumor effect, we are reasonably confident that infusion of more killer cells of one or another type may increase the antitumor efficacy overall, however, may also increase the level of cytokine secretion and possible side effects in association.

In conclusion, our study compares the NK-cell engager BK1 (BCMA×CD16) with the T-cell engager BT1 (BCMA×CD3) with the same IgG-like configuration. BK1 activates NK cells, while BT1 activates T cells, both of which elicit significant antitumor effects *in vivo*. Furthermore, BK1 elicits stronger antitumor efficacy than BT1 when the numbers of NK cells and T cells are equal. In addition, BK1 induces less proinflammatory cytokine production than BT1. Combined treatment with BT1 and BK1 shows better antitumor efficacy than either treatment alone. Surprisingly, BK1 also reduced proinflammatory cytokine production by BT1 in the combined treatment. Our study points out the advantages of NK cell engager over T-cell engager: use of NK cell engager alone may elicit stronger antitumor efficacy with less proinflammatory cytokine production, and the combined usage of NK cell engager with T-cell engager not only further improves the antitumor effect but also lowers cytokine release from T-cell engager, suggesting that NK cell engager has been previously underestimated and is a promising option in treating tumors.

## Data availability statement

The datasets presented in this study can be found in online repositories. The names of the repository/repositories and accession number(s) can be found below: https://www.uniprot.org/, Q02223 https://www.uniprot.org/, P07766 https://www.uniprot.org/, P08637 https://pubmed.ncbi.nlm.nih.go,10.1084/jem.175.1.217 https://pubmed.ncbi.nlm.nih.go, US9273141B2.

## Ethics statement

The animal study was reviewed and approved by Ethics Committee of the University of Science and Technology of China.

## Author contributions

XX, YC, HS, ZT designed the study. YC, RS, YF, YZ performed the experiments and analyses. RS and XZ provided advice. XX, HS, ZT wrote and revised the manuscript. HS, ZT, RS and XZ supervised the study. HS and ZT critically reviewed the manuscript. All authors contributed to the article and approved the submitted version.
