# Corrigendum: Bispecific NK-cell engager targeting BCMA elicits stronger antitumor effects and produces less proinflammatory cytokines than T-cell engager

**DOI:** 10.3389/fimmu.2024.1365027

**Published:** 2024-01-16

**Authors:** Xinghui Xiao, Ying Cheng, Xiaodong Zheng, Yuhang Fang, Yu Zhang, Rui Sun, Zhigang Tian, Haoyu Sun

**Affiliations:** ^1^ Hefei National Research Center for Physical Sciences at Microscale, The CAS Key Laboratory of Innate Immunity and Chronic Disease, School of Basic Medical Sciences, Division of Life Sciences and Medicine, University of Science and Technology of China, Hefei, China; ^2^ Institute of Immunology, University of Science and Technology of China, Hefei, China; ^3^ Hefei TG ImmunoPharma Corporation Limited, Hefei, China

**Keywords:** NK-cell engager, T-cell engager, tumor immunotherapy, antitumor, proinflammatory cytokines

In the published article, there was an error in the funding statement for the National Key R&D Program of China (2019YFA0508502/3, 2018YFA0507403); Natural Science Foundation of China (8222053, 81972679); Natural Science Foundation of Anhui Province (2008085MH252). The correct Funding statement appears below.

