# Supplementary material for: Bispecific NK-cell engager targeting BCMA elicits stronger antitumor effects and produces less proinflammatory cytokines than T-cell engager
Source: Front Immunol. 2023 Apr 11;14:1113303. doi: 10.3389/fimmu.2023.1113303 (PMC10126364; doi:10.3389/fimmu.2023.1113303)
Supplement: Supplementary file 1 [file DataSheet_1.pdf]

Supplemental Information

Bispecific NK-cell engager targeting BCMA elicits stronger antitumor effects and produces less proinflammatory cytokines than T-cell engager

Xinghui Xiao[1,2], Ying Cheng[1,2], Xiaodong Zheng[1,2], Yuhang Fang[1,2], Yu Zhang[1,2], Rui Sun[1,2], Zhigang Tian [1,2,3,*], Haoyu Sun [1,2,*]

***Correspondence:**

*Author for correspondence: Dr. Haoyu Sun and Dr. Zhigang Tian, Institute of Immunology and CAS Key Laboratory of Innate Immunity and Chronic Disease, School of Basic Medical Sciences, Division of Life Sciences and Medicine, University of Science and Technology of China, 443 Huangshan Road, Hefei, Anhui 230027, China; Tel: 86-551-6360-7377; Fax: 86-551-6360-6783; E-mail: haoyusun@ustc.edu.cn (H Sun), tzg@ustc.edu.cn (Z Tian)

This pdf contains

Supplemental Figures and Legends

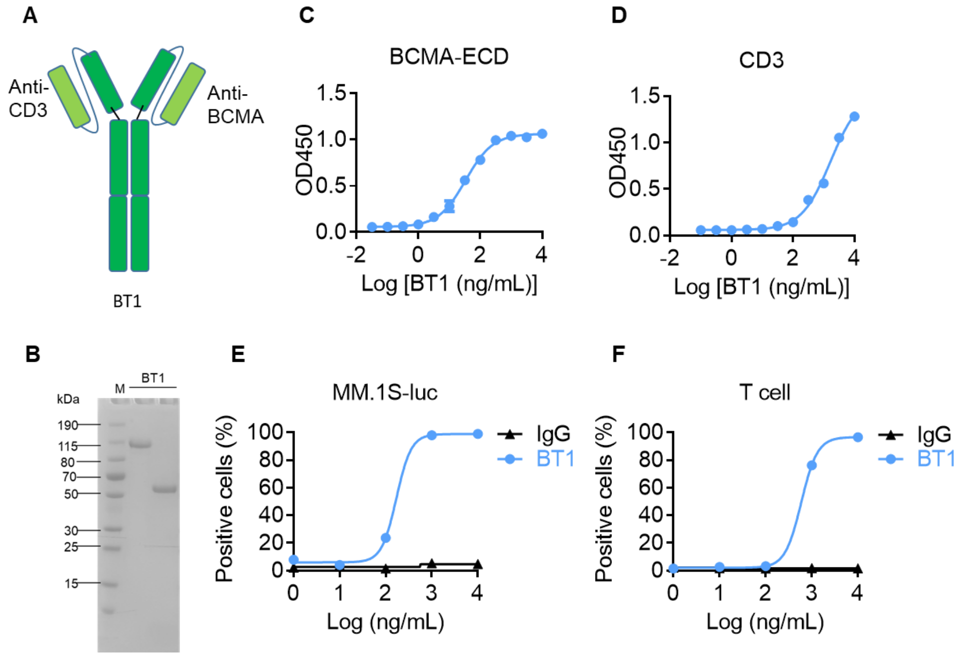

Figure S1. Structural features and binding affinity of BT1 (BCMA×CD3) bispecific antibody. (A) Schematic representation of BT1 (CD3×BCMA), a two-arm IgG1-based human antibody. (B) The molecular weight and purity of BT1 were determined by SDS-PAGE. (C) Binding specificity of BT1 with BCMA extracellular domain protein. (D) Binding specificity of BT1 with CD3 protein. (E) Binding specificity of BT1 with $BCMA^+$ MM.1S-luc cell. BT1 was labeled with Alexa Fluor 647 fluorescein and incubated together with a $BCMA^+$ MM.1S-luc cells at indicated concentrations followed by flow cytometric analysis. (F) Binding specificity of BT1 with $CD3^+$ T cells. BT1 was labeled with Alexa Fluor 647 fluorescein and incubated together with $CD3^+$ T cells at indicated concentrations followed by flow cytometric analysis.

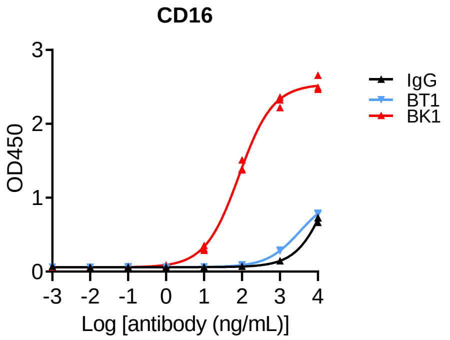

Figure S2. Binding ability of BK1 and BT1 to CD16 protein.

Binding specificity of IgG, BT1 and BK1 to CD16 respectively.

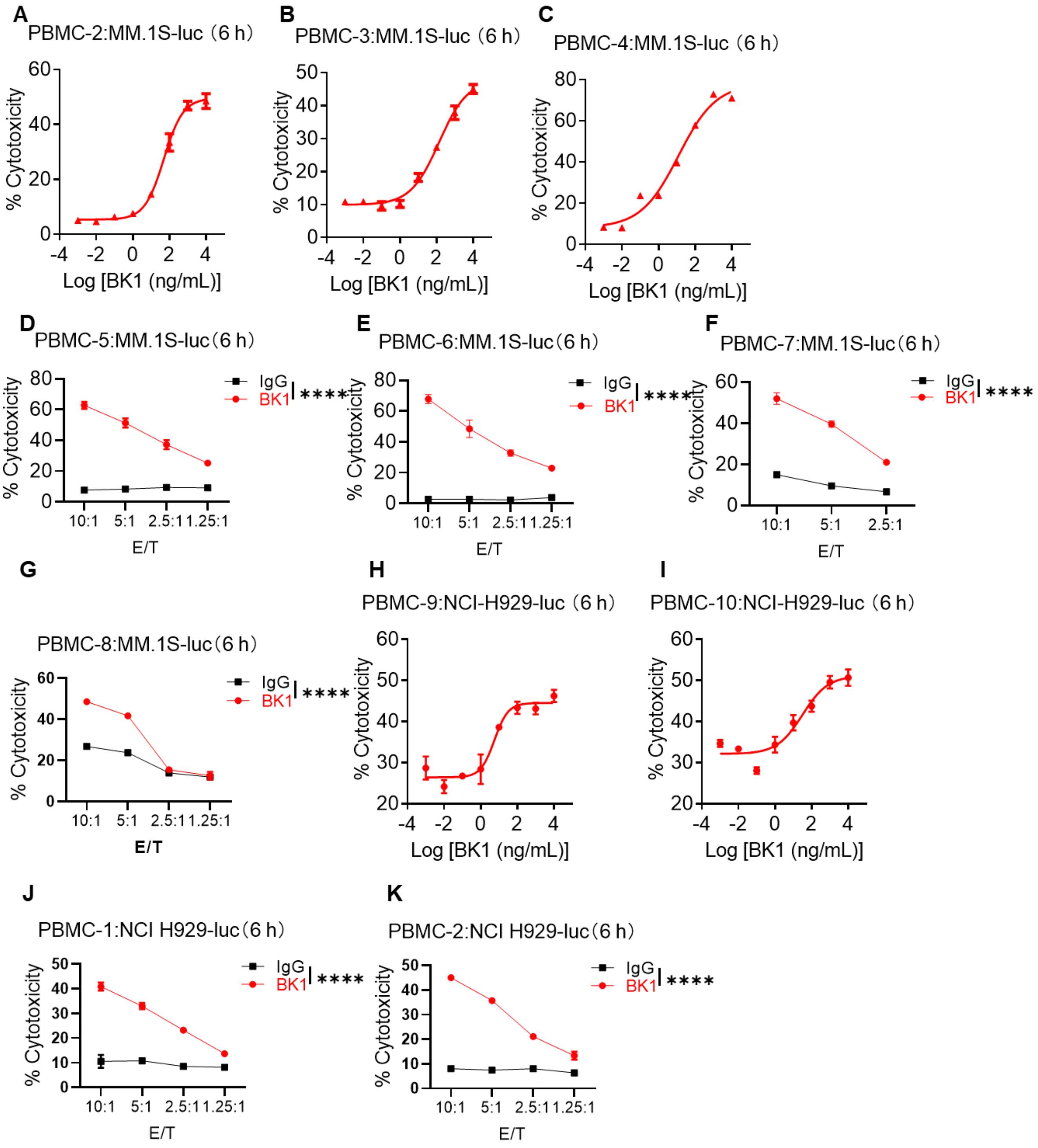

Figure S3 BK1 (BCMA×CD16A) specifically kills BCMA^{+} target cells

(A-C) Percentage of tumor cell lysis after coculturing MM.1S-luc cells with PBMCs in the presence of BK1 at an E:T ratio of 10:1 for 6 h. (D-G) Percentage of tumor cell lysis after coculturing MM.1S-luc cells with PBMCs in the presence of BK1 (1 μg/mL) at the indicated E:T ratios for 6 h. (H-I) Percentage of tumor cell lysis after coculturing NCI H929-luc cells with PBMCs in the presence of BK1 at an E:T ratio of 10:1 for 6 h. (J-K) Percentage of tumor cell lysis after coculturing NCI H929-luc cells with PBMCs in the presence of BK1 (1 μg/mL) at the indicated E:T ratios for 6 h. PBMCs were obtained from a different donor for each independent experiment. Data were analyzed by Student's t test or 2-way analysis of variance. ****P < 0.0001.

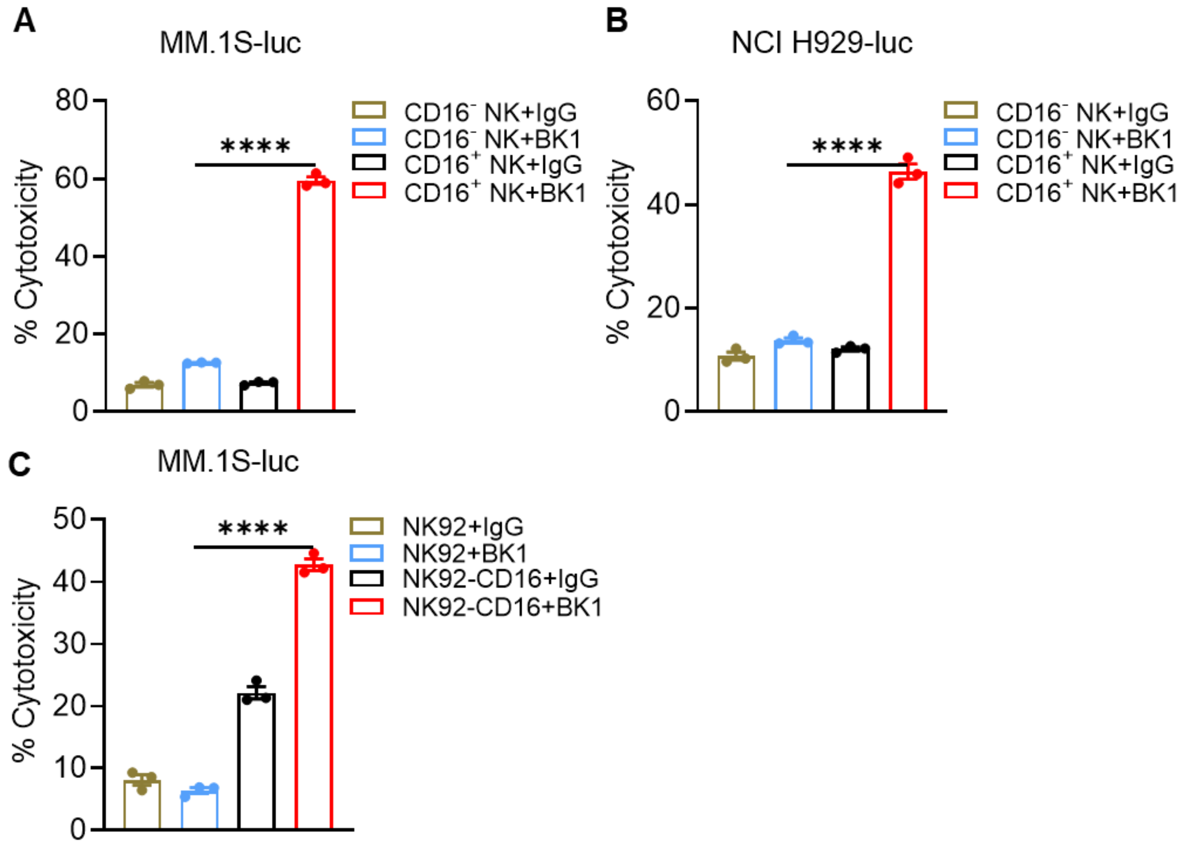

Figure S4. BK1 (BCMA×CD16A) specifically acts on $CD16^+$ NK cells.

(A) Percentage of tumor cell lysis after co-culturing MM.1S-luc cells with $CD16^+$ or $CD16^-$ NK cells in the presence of IgG or BK1 at an E:T ratio of 2:1 for 6 h. (B) Percentage of tumor cell lysis after co-culturing NCI H929-luc cells with $CD16^+$ or $CD16^-$ NK cells in the presence of IgG or BK1 at an E:T ratio of 2:1 for 6 h. (C) Percentage of tumor cell lysis after co-culturing MM.1S-luc cells with NK92-CD16 cells or NK92 cells in the presence of IgG or BK1 at an E:T ratio of 2:1 for 6 h. Data were analyzed by Student's t test. ****$P < 0.0001$.

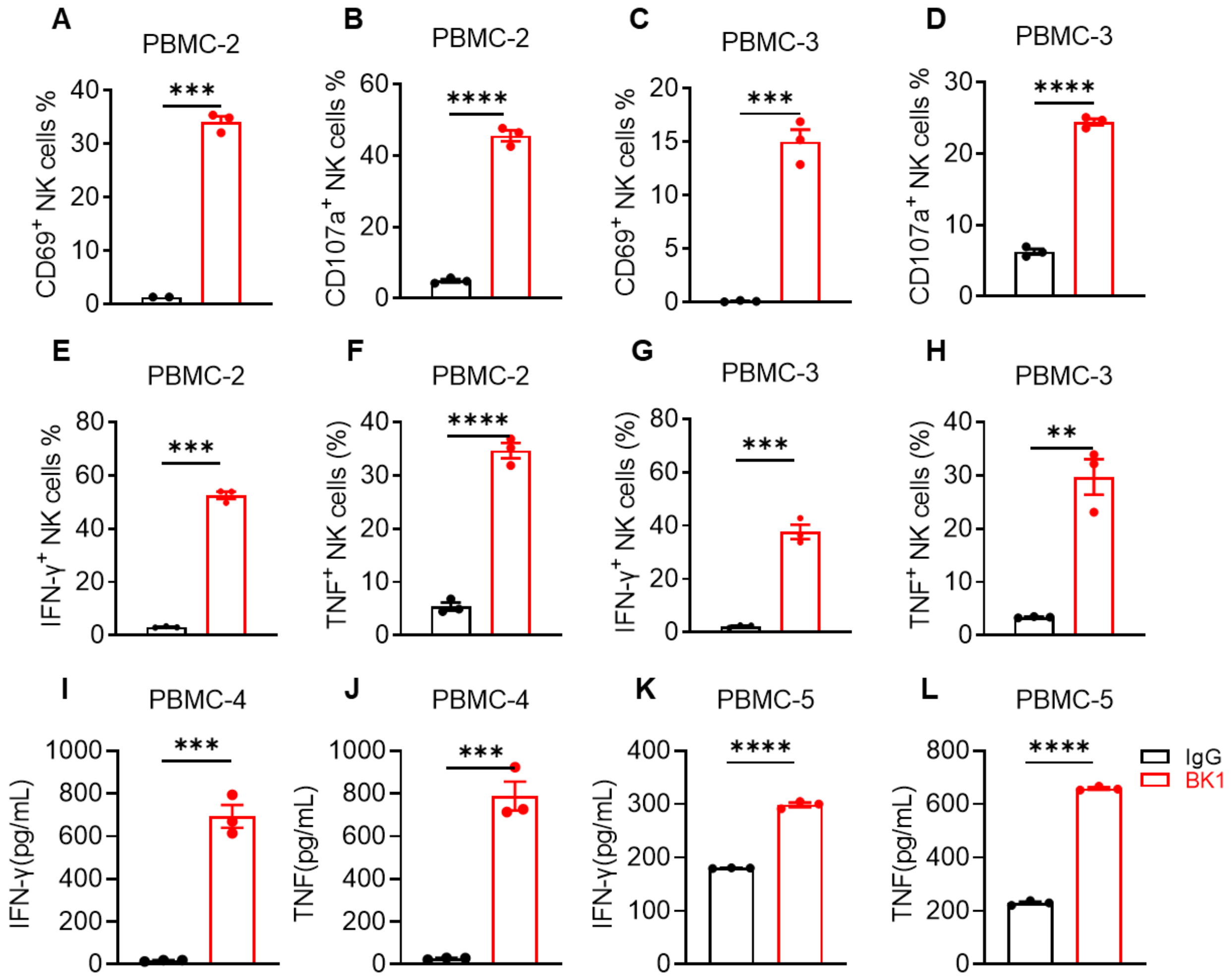

Figure S5. BK1 (BCMA×CD16A) activates NK cells.

(A-H) PBMCs were cocultured with MM.1S-luc cells at an E:T ratio of 10:1 in the presence of IgG (1 μg/mL) or BK1 (1 μg/mL) for 6 h, and $CD3^{-}CD56^{+}$ NK cells were assessed for the expression of CD69,CD107a, IFN-γ,TNF by flow cytometry. (I-L) PBMCs were cocultured with MM.1S-luc cells at an E:T ratio of 10:1 in the presence of IgG (1 μg/mL) or BK1 (1 μg/mL) for 6 h, and the levels of secreted IFN-γ and TNF in the supernatants were assessed using a CBA Kit. Data were analyzed by Student's t test, ***P < 0.001, ****P < 0.0001.

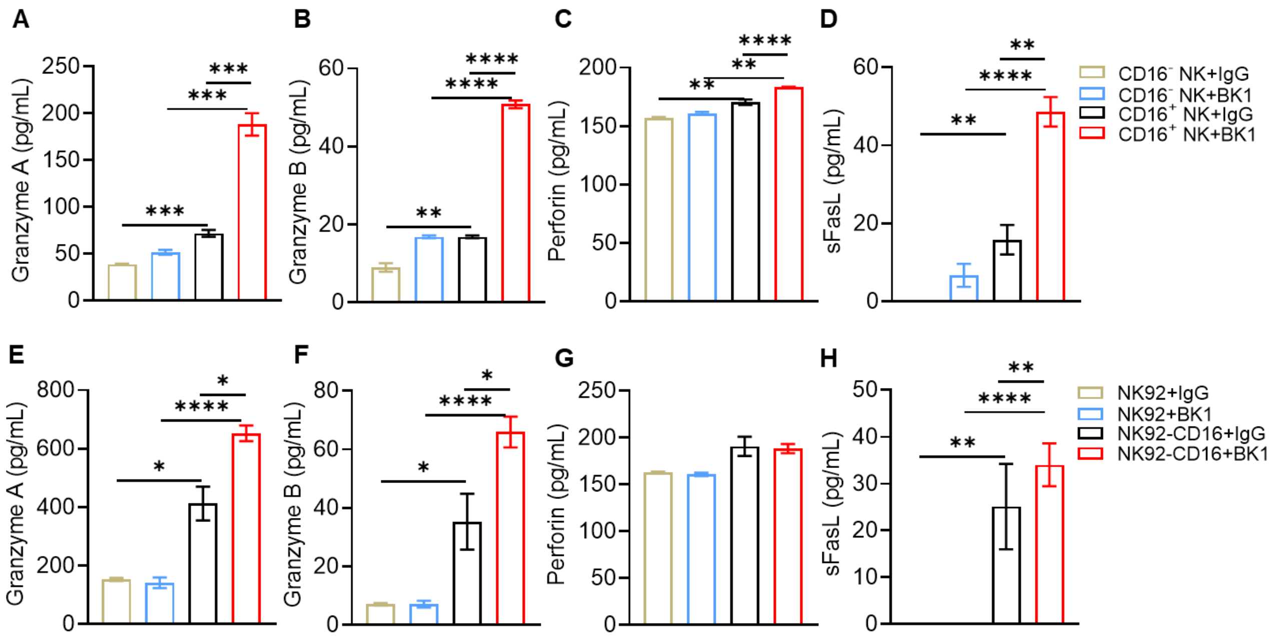

Figure S6. BK1 (BCMA×CD16A) enhances the cytokine secretion by CD16^{+} NK cells.

(A-D) CD16^{+} NK cells or CD16^{-} NK cells were co-cultured with MM.1S-luc cells at an E:T cell ratio of 2:1 in the presence of IgG (1µg/mL) or BK1 (1µg/mL) for 6 h, and the supernatants were assessed for levels of secreted (A) Granzyme A, (B) Granzyme B, (C) Perforin, and (D) sFasL using LEGENDplex™ Human CD8/NK Panel. (E-H) NK92-CD16 cells or NK92 cells were co-cultured with MM.1S-luc cells at an E:T cell ratio of 2:1 in the presence of IgG (1µg/mL) or BK1 (1µg/mL) for 6 h, and the supernatants were assessed for levels of secreted (E) Granzyme A, (F) Granzyme B, (G) Perforin and (H) sFasL using LEGENDplex™ Human CD8/NK Panel. Data were analyzed by Student's t test. *P < 0.05; **P < 0.01; ***P < 0.001; ****P < 0.0001.

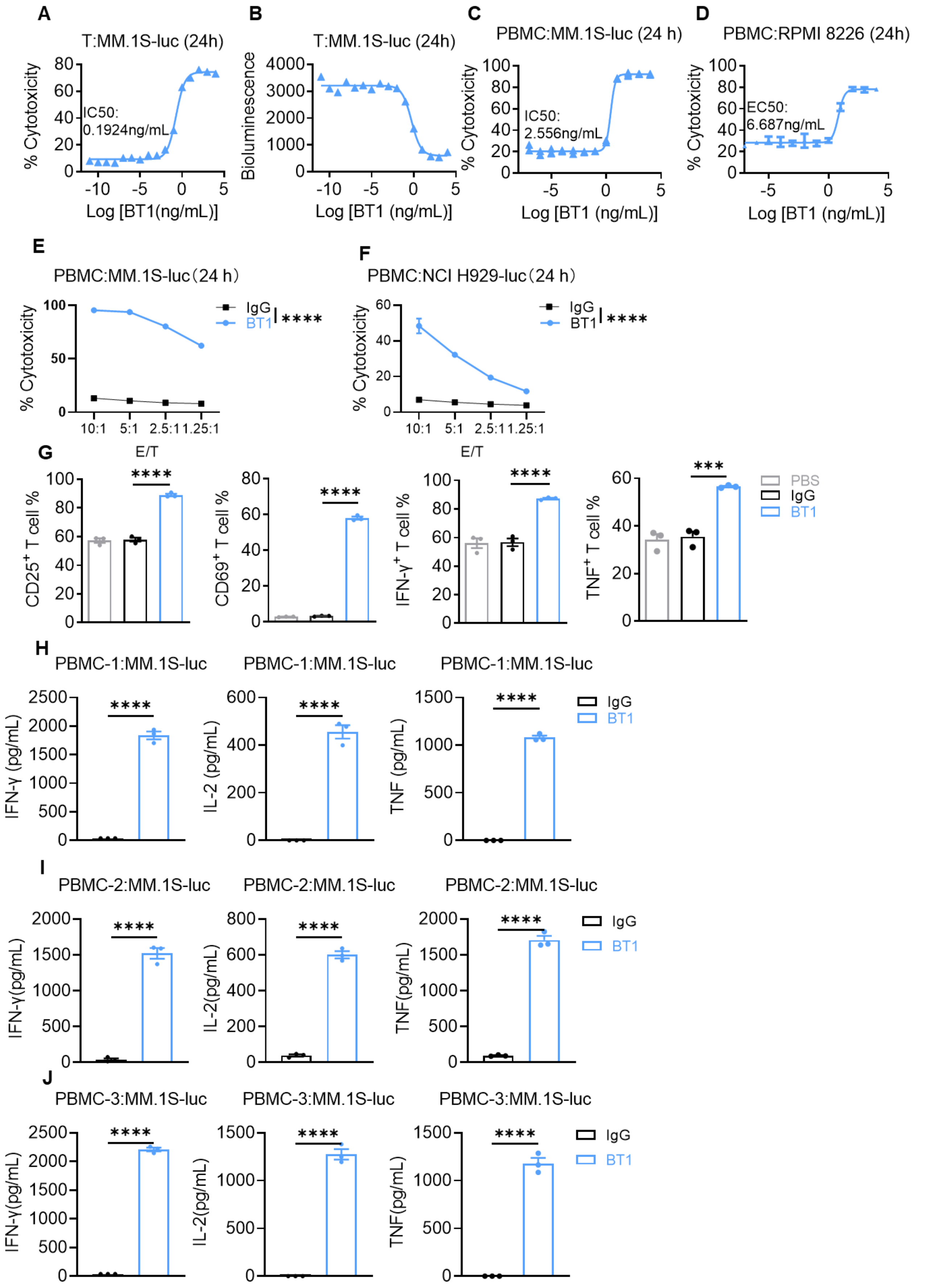

Figure S7. BT1 (BCMA×CD3) specifically kills BCMA$^+$ target cells and activates T cells. (A)

Percentage of tumor cell lysis after co-culturing MM.1S-luc cells with purified T cells in the presence of BT1 at an E:T ratio of 2:1 for 24 h. (B) The bioluminescence of tumor cells after co-culturing MM.1S-luc cells with purified T cells in the presence of BT1 at an E:T ratio of 2:1 for 24 h. (C) Percentage of tumor cell lysis after co-culturing MM.1S-luc cells with PBMCs in the presence of BT1 at an E:T ratio of 10:1 for 24 h. (D) Percentage of tumor cell lysis after co-culturing RPMI 8226 cells with PBMCs in the presence of BT1 at an E:T ratio of 10:1 for 24 h. (E, F) Percentage of tumor cell lysis after coculturing (E) MM.1S-luc cells or (F) NCI H929-luc cells with PBMCs in the presence of IgG (1 μg/mL) or BT1 (1 μg/mL) at the indicated E:T ratios for 24 h. (G) PBMCs were co-cultured with MM.1S-luc cells at an E:T ratio of 10:1 in the presence of IgG (1μg/mL) or BT1 (1μg/mL) for 24 h, and $CD3^{+}CD56^{-}$ T cells were assessed for expression of CD25, CD69, IFN-γ and TNF-α by flow cytometry. (H-J) PBMCs from different people were co-cultured with MM.1S-luc cells at an E:T cell ratio of 10:1 in the presence of BT1(1μg/mL) or IgG (1μg/mL) for 24 h, and the supernatants were assessed for levels of secreted IFN-γ, TNF-α, IL-2 and IL-6 using CBA Kit. Data were analyzed by Student's t test. *$P < 0.05$; **$P < 0.01$; ***$P < 0.001$; ****$P < 0.0001$.

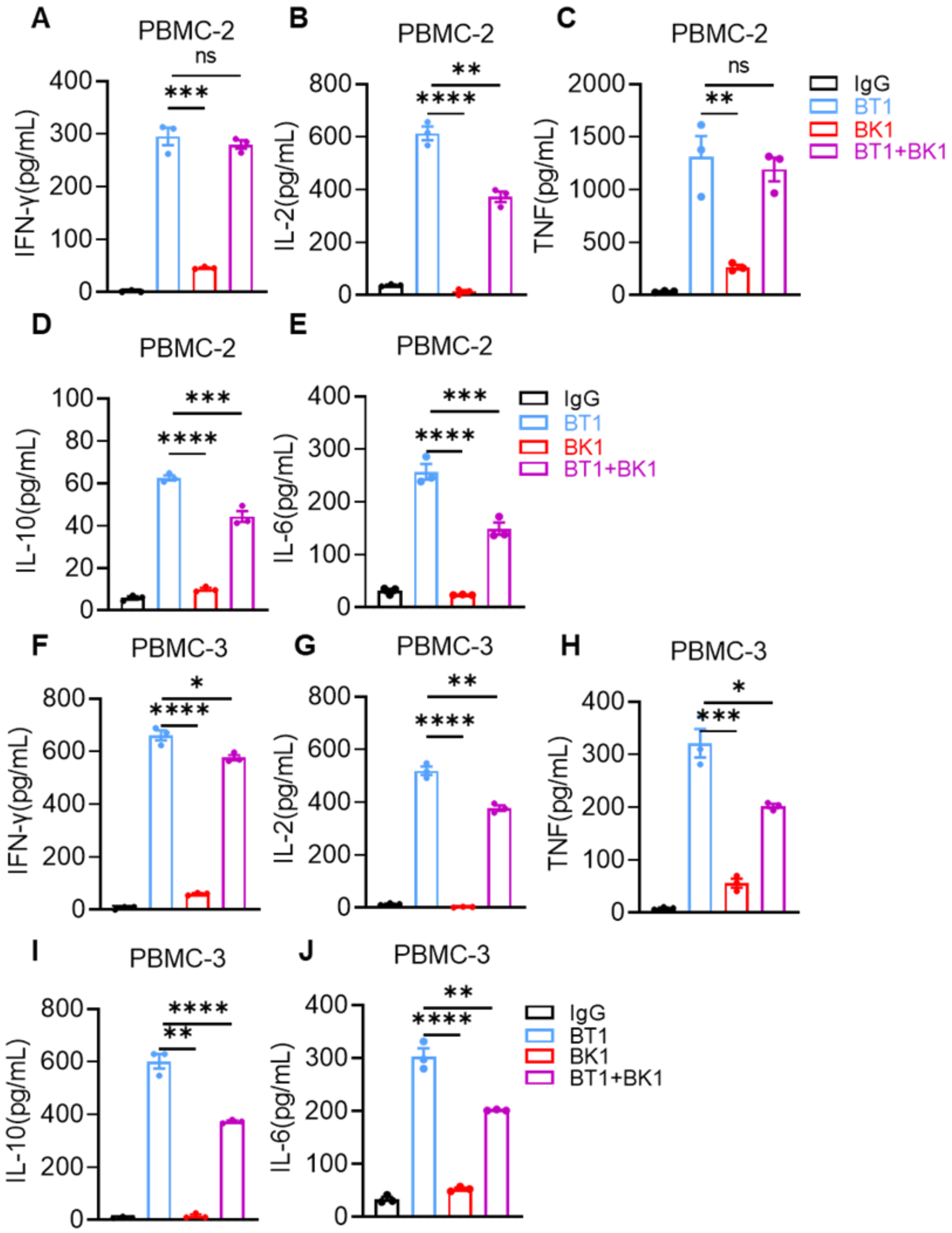

Figure S8 BK1 induces less pro-inflammatory cytokine production and reduces the cytokine secretion by BT1 in combined treatment.

(A-E) PBMCs were cocultured with MM.1S-luc cells at an E:T ratio of 10:1 in the presence of IgG, BT1, BK1 or BT1 combined with BK1 (1 μg/mL) for 24 h, and the levels of secreted (A) IFN-γ, (B) IL-2, (C) TNF, (D) IL-10 and (E) IL-6 in the supernatants were assessed using a CBA Kit.

(F-J) PBMCs (different donor) were cocultured with MM.1S-luc cells at an E:T ratio of 10:1 in the presence of IgG, BT1, BK1 or BT1 combined with BK1 (1 μg/mL) for 24 h, and the levels of secreted (F) IFN-γ, (G) IL-2, (H) TNF, (I) IL-10 and (J) IL-6 in the supernatants were assessed using a CBA Kit. Data were analyzed by Student's t test. *$P < 0.05$; **$P < 0.01$; ***$P < 0.001$; ****$P < 0.0001$.

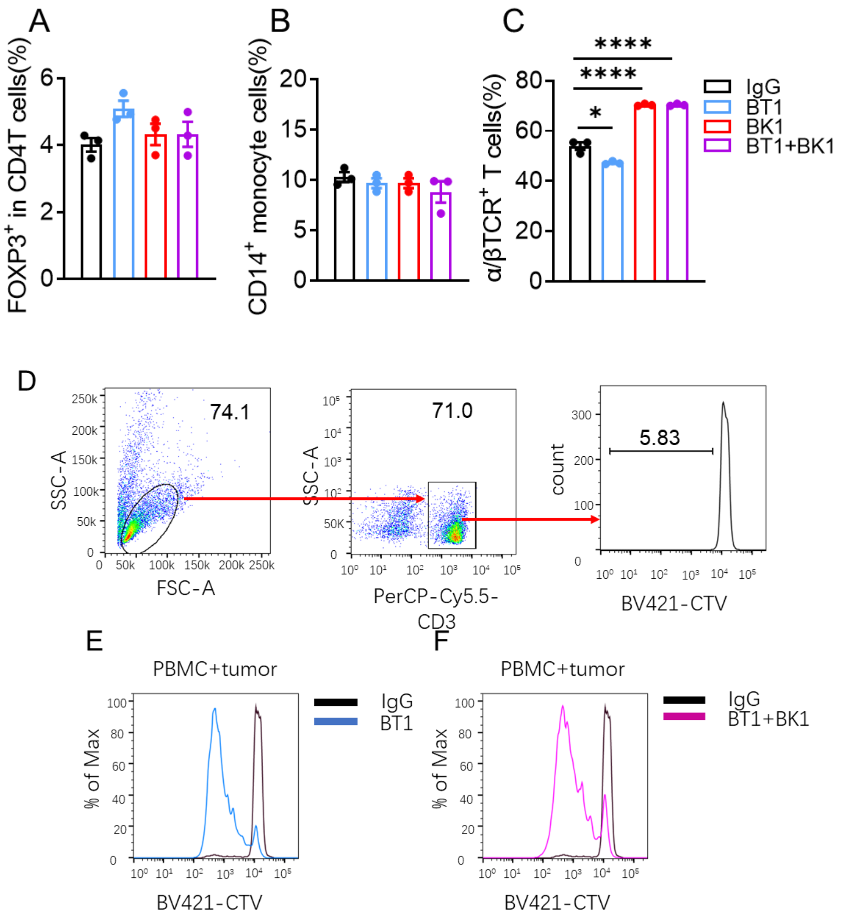

Figure S9. BK1 enhances the proliferation of T cells.

(A-C) PBMCs were co-cultured with MM.1S-luc cells at an E:T ratio of 10:1 in the presence of IgG (1μg/mL), BT1 (1μg/mL), BK1 or BT1combined with BK1 for 24 h, and the percentage of (A) α/β TCR^{+} T cells, (B) α/βTCR^{+}CD4^{+}Foxp3^{+} T cells or (C) CD14^{+} monocyte cells was assessed by flow cytometry. (D-F) CTV^{+} PBMCs were co-cultured with MM.1S-luc cells at an E:T ratio of 1:1 in the presence of IgG (1μg/mL), BT1 (1μg/mL) or BT1 (1μg/mL) combined with BK1 (1μg/mL) for 3 d, and CTV^{+}CD3^{+} T cells were assessed by flow cytometry. (D) Flow cytometry gating strategy. (E) CTV^{+}CD3^{+} T cells were assessed in IgG and BT1 groups. (F) CTV^{+}CD3^{+} T cells were assessed in IgG and BT1+BK1 groups.
